# Women in ASHE

**DOI:** 10.1017/ash.2025.34

**Published:** 2025-02-24

**Authors:** Louise-Marie Dembry

**Affiliations:** Yale University School of Medicine, Yale School of Public Health, New Haven, CT, USA

Dr. Dembry is Professor of Medicine, Infectious Diseases and Epidemiology at Yale University. She was the Yale-New Haven Hospital (YNHH) Associate Director and Director of Hospital Epidemiology from 1993 to 2015. She was the Director of Hospital Epidemiology for the VA CT Healthcare System 1993–2001 and 2015–2022. She has held multiple hospital leadership positions at both YNHH and the VA CT Healthcare System. She has served on multiple Society for Healthcare Epidemiology of America (SHEA) committees and work groups. She was President of SHEA and Board Chairperson in 2016 and served as the SHEA liaison to CDC’s Healthcare Infection Control Practices Advisory Committee (HICPAC) 2017–2021. She was a voting member of the Connecticut Healthcare Associated Infection Advisory Committee appointed by the Commissioner of Public Health 2006–2024 and is a member of the CT DPH HAI Multidisciplinary Antimicrobial Resistance Advisory Group. Her main interests include (1) the prevention and control of transmission of antibiotic-resistant organisms in healthcare settings, (2) patient safety, (3) healthcare facility preparedness for high consequence infections, and (4) training and education for hospital epidemiology and public health emergencies.

1. At your IDWeek 2024 SHEA lectureship, you spoke of your career journey to leadership in healthcare epidemiology. In your experience, how has the field evolved over the past few decades, particularly in response to emerging infectious diseases?

I would start by going back a little bit further to the role of public reporting before I talk about emerging infectious diseases, which has increased our visibility as a profession. Looking back, I would say the field started gaining more attention in the last 20 to 25 years with the rise of public reporting of healthcare-associated infection (HAI) rates and pay for performance reimbursement models. In the early 2000s, there was a lot of discussion and tremendous push from patient advocate groups to implement public reporting of HAIs which did eventually become a requirement. I think this made us rise in the level of importance in our healthcare systems, hospitals, and facilities. For the administrators, this was now something they had to give attention to. Public reporting mandates made them recognize not only who we were, but our important role in addressing the issues and they needed us. This was a big change for the field, it heralded an era of more transparency.

Now, layer emerging infections onto that and our role in preparedness and response. Back in the 1990s, I dealt with a viral hemorrhagic fever research laboratory exposure at the Yale School of Public Health and was brought into the response that was led by the CDC. Once the immediate threat was resolved, the event melted into the background and people forgot about it. Then in 2001, I became a point person in the Yale-New Haven Hospital (YNHH) response to the anthrax mailings after a case was diagnosed in a Connecticut resident. Healthcare facilities needed to plan realistically how they were going to respond in a way that they had not done before. That drew my career into public health and emergency preparedness, and I was appointed to the new role of Yale-New Haven Health System medical director for emergency preparedness. The teamwork needed to manage this complex role was eye opening and a challenge I relished. It also garnered the attention of leadership, something that is critical for healthcare epidemiologists.

It was an opportunity to lead and show the value of the healthcare epidemiologist that extended beyond what one might call traditional infection prevention. There was an increased awareness of what we’re capable of doing. Being part of the response to emerging infectious diseases helped propel healthcare epidemiologists to the forefront. Now, it’s not what we do every day, but there have been enough events in the last 25 years, like SARS in 2003 then the H1N1 influenza pandemic in 2009, followed by MERS, measles, Ebola, and COVID-19 that have given us ample opportunities to showcase our unique skills and talents. We are always thinking about preparedness so that we’re ready when administrators and leadership come asking “what is it we’re supposed to do?” Emerging infectious diseases preparedness and HAI public reporting highlight our importance, and we should leverage that attention to strengthen and grow our programs.

There is a quote that I use in my talks about high consequence infections from Rahm Emanuel that is a take on Winston Churchill’s quote about never letting a good crisis go to waste. Rahm Emanuel said “You never want a serious crisis to go to waste. And what I mean by that is an opportunity to do things that you think you could not do before.” I think that the way I look at our role in the last 25 years in responding to emerging infectious diseases, has not only pushed us as individuals, but has shown that we can push our institutions and our facilities to do things we never thought possible.

1. What are some of the important skills or additional training you acquired along the way, and how did they help grow your career? Do you recommend these to others?

It’s hard to know what skills we’ve acquired along the way, because there are no benchmarks, but I think one of the skills is being able to manage people and programs. Many of us end up in roles where we must manage people, which is not something that we’re taught in our training as physicians. I try to understand every individual on the team, what their motives are, what they want to accomplish, and then try to support them in doing that, and how they can support the team and the program. Part of that is having good listening skills, which I will admit I’m still working on. I think back to what somebody said to me once, which is that people just want to be heard and truly listened to. Another skill is understanding the business and operations of a healthcare organization. I went to business school and got my MBA in 2007 because I felt I wasn’t being taken seriously for the business skills I acquired on the job because I didn’t have an MBA or an MHA. I recognized that administrators were never going to learn my language, so I had to learn theirs for them to listen and eventually come to trust me. Not that I am suggesting that everybody get an MBA degree, but it is important for us to be able to communicate with administrators, who are mostly non-clinicians and see us as a little bit of an enigma. By speaking their language and understanding their priorities, we can dialog more effectively. I chose to do a general MBA because I feel that healthcare can learn a lot from business, and we shouldn’t silo ourselves and say we are too different to benefit from that fund of knowledge and experience. Part of that is to learn to be an effective lobbyist, another skill that I think is beneficial for healthcare epidemiologists to have.

1. What have been the biggest barriers to implementing positive change in your healthcare environment? Were there certain initiatives with too many hurdles that you had to abandon?

I think I encountered the biggest barriers when we could not get the attention or support we needed to either get important initiatives started, sustain them or take them to the next level. I spent a lot of time “lobbying” all levels of leadership as well as frontline staff. We are trying to make our priorities become somebody else’s priority because they have many of their own. You must have the support of the people implementing these initiatives and the support of leadership, because they can make it important for others. If leadership says, “this is one of our top 10 priorities,” that helps us. Another barrier beyond initial implementation is sustaining gains and improvements and keeping people engaged. Once we see improvements, we tend to move on to the next thing, and then practice slides back to what it was. Being able to sustain improvements in a milieu where there are a lot of competing priorities is a challenge.

There were initiatives I had to abandon because I could not get the needed traction and people lost interest. However, I learned that if others had ownership of the initiative that I tended to get more engagement. For instance, if there is a team that comes together to decrease healthcare-acquired *C. difficile* infections, I don’t think it is a given that infection prevention should lead that team. We are the content experts and understand implementation science, we provide guidance and interpret the literature, but the effort may best be led by others who have more control and ownership over the process itself. It becomes harder to abandon something one owns and feels responsible for its success or failure.

1. What are some of your biggest career wins and what was the key to their success?

Probably my biggest career win was making hand hygiene a top priority for YNHH. I had been trying to get traction on hand hygiene since my arrival at YNHH in 1993 when I was tasked to manage the prevention of transmission of vancomycin-resistant enterococci (VRE). Our hand hygiene compliance rates were abysmal, and I couldn’t seem to get traction until the new chief of staff (who was my boss) supported my hand hygiene efforts in the early 2000s. With his support, we were an early adopter of alcohol-based hand rub which helped overcome a big barrier. Sinks were few and far between and that was often cited as the reason for poot hand hygiene compliance. The chief of staff made it his mission to lead by example and bring everyone along with him, he was adamant that the status quo was not acceptable and would not be tolerated. Hand hygiene became a multi-year business plan priority and was on every manager’s performance review. Compliance with hand hygiene also became a metric of the hospital incentive program that all employees participated in, like readmissions and early discharges. So, everybody had a stake in the game, no matter their hospital role. To me that was a career win, and I learned the value of partnering with the right leader. We took hand hygiene compliance from an abysmal 20% compliance up to ∼80%. We were able to sustain that gain however we were not able to increase compliance further. As we know, performing hand hygiene correctly and consistently positively affects every patient, staff member, and visitor and makes the hospital safer for everybody. One of the things I enjoy most about healthcare epidemiology is the impact our efforts have on a population, not only one individual at a time.

The COVID-19 pandemic was also a type of career win for me. The many years I spent working on high consequence infection preparedness and having been involved in SARS in 2003 working at a Toronto hospital, the 2009 H1N1 influenza pandemic, and the 2014–2015 Ebola Virus Disease outbreak came to fruition during the rapidly evolving COVID-19 pandemic. By the time January 2020 rolled around, I had accumulated lots of experience that positioned me to guide the facility response and to be an effective consultant to the incident commander. I knew it was crucial to have leadership’s trust but when I felt they were slow to understand the gravity of what was quickly coming, I leveraged other relationships rather than wait while I continued to lobby leadership to take the imminent threat seriously. Our approach and policies were not always popular with staff, patients and visitors but every decision was based on “safety first.” We were recognized for having one of the lowest healthcare-associated COVID-19 infection rates in the VA system. I was also finally able to get resources to grow a very small healthcare epidemiology/infection prevention team into a robust program that became part of my succession plan and remains in place today. Remember, never let a good crisis go to waste!

1. Can you tell us about important mentors who shaped your career?

As I mentioned in my lectureship talk, I became interested in healthcare epidemiology during fellowship from an infectious diseases (ID) attending who asked me if I knew anything about the behind-the-scenes processes in the dialysis unit. That really spoke to my desire to understand how things work in the background, I was hooked from that point forward. This was in the early 1990s—healthcare epidemiology wasn’t as robust of a career path and there were few mentors. Where I trained, the closest person to being a healthcare epidemiologist was Dr. Mark Zervos. He was more of a lab researcher at the time but had been at Yale as the YNHH Associate Hospital Epidemiologist under Dr. Walter Hierholzer in the 1980’s. So, I worked in Dr. Zervos’ lab for 2 years as part of my three-year ID research fellowship. I worked on VRE, Acinetobacter, and Candida investigating molecular dynamics of transmission and had the opportunity to do some cluster investigations. Dr. Zervos supported sending me to training courses and helped fuel my passion for epidemiology. Through his connection to Yale, I was invited to interview for the YNHH Associate Hospital Epidemiologist and Director Antimicrobial Stewardship position in 1993 that also included being the VA Hospital Epidemiologist—it was a big position, one for which I had no training but had a burning desire to pursue. I have no doubt that my personal connection with Dr. Zervos helped me get the position and launch my career. Another significant mentor was my fellowship program director Dr. Donald Levine. He celebrated my successes and pushed me to challenge myself especially when I doubted myself and my abilities. When I first came to Yale, another instrumental mentor was Dr. Vincent Andriole who was not an epidemiologist, but a well-known infectious diseases physician. He took me under his wing, he expected a lot from me, and I feared disappointing him but at the same time I always knew he had confidence in my abilities which was a needed boost when I was unsure of myself. He guided me through the morass of academia and gave me opportunities that strengthened my chances for academic advancement. A very proud moment was when I called to tell him that I had finally been promoted to full professor in 2011 after several tries, and his response was “you made it, kid.”

Then there are the many mentors and sponsors I had, and still have, related to my healthcare epidemiology career. There are too many to name and if I tried, I would inadvertently forget to name someone who was instrumental in guiding me. I only hope every one of my mentors knows who they are, how important they’ve been, and how much I value their mentoring. There are the mentors who gave me my first breaks to participate in the Society for Healthcare Epidemiology of America (SHEA) by inviting me to serve on committees and task forces starting in 1997. They encouraged me to run for the SHEA Board, which I did the first time in 2009 for the position of Treasurer and later for Vice President in 2013 and again when I ran successfully in 2014. I worked with five different SHEA executive directors during my career, each was a great teacher and partner as I learned about the world of a professional society and advocating for our profession. There are the mentors who are part of my support network, always available to listen and provide suggestions at periods of transition in my career when I was at a crossroads and not sure which path to take. And I would be remiss if I did not acknowledge the many infection preventionists I’ve worked with. I learned so much about navigating hospital politics from them, such that when I train new infection preventionists I make sure to share those invaluable lessons. In my last years at the VA, one of the infection preventionists I trained ultimately took on the role of infection prevention program manager. Shortly before I stepped down at the end 2022, he stated “I channeled my inner Louise” when describing how he handled a difficult discussion. I responded, “My work here is done!”

1. You’ve also mentored many professionals, especially woman. How have you personally benefited from serving in this role?

I’ve had female and male mentors and have mentored both women and men. I think women gravitate to women mentors as we are more likely to have shared experiences in the academic setting. We all have setbacks and disappointments and I’m keen about sharing mine. My mentoring role reminds me how much I’ve accomplished and the many connections I have as a result. I am now able to help others, actively sponsor them, and pay it forward as my way of thanking my mentors. I find that women are more likely to suffer from imposter syndrome, or maybe they are just more likely to articulate it than men, so celebrating their accomplishments is important. Mentoring is also a good time to practice listening skills, which like I said, I’m still working on. Mentoring is less about mapping out a career plan for somebody, and more about listening to them and encouraging them to listen to themselves about what they really want, what they are passionate about and acknowledge that this may change over time and that’s ok. I was encouraged by one of my early mentors to check in with myself every 5 years and ask “is it time to make a change? Why or why not?” Pushing mentees to ask these questions also pushes me to do the same, especially in the face of a setback…“Is it time to be open to something else or should I persevere? What do I want to accomplish professionally and personally going forward?” I learned from my mentors that we don’t necessarily see ourselves as others see us. As women, we tend to downplay our achievements, second-guess ourselves or perceive a setback to mean we are not capable enough. Mentoring others has given me a different perspective on my career. I am more likely now to see setbacks as opportunities and am more willing to celebrate achievements as something well deserved that I earned.

1. What are some career obstacles you faced as a woman in medicine? Was there a particular “glass ceiling” that you felt was inaccessible to you as a woman? What advice would you have for your younger self navigating those challenges?

When I interviewed for the Yale and YNHH position in 1993, one of the interviewers told me that I had a better chance of getting the position as a woman since Yale was making an effort to recruit more women faculty but once hired, I would have a harder time than would a man. I’ve often been reminded of that statement during my career. I didn’t feel that my male colleagues who were in similar type roles heading a department, or a program had to prove themselves in the same way. It seemed that they came to the role with the assumption that they could do it while I had to prove myself time and again, especially during a crisis or whenever there was a change in leadership.

I can’t say that there was a “glass ceiling” that was inaccessible to me as a woman since I did not aspire to move into other positions. However, I did experience issues related to pay parity with men. After being at Yale for ∼5 years, I became aware that I was paid a lot less than my male counterparts and there wasn’t really anything I could do about it as an individual. A group of senior women faculty started to speak out and push for change which although it came slowly there is now more transparency. Throughout my career, some of my mentors would say to me, “you could go somewhere else and be appreciated and valued for your skills and experience” during times when I was uncertain of being able to hold onto my position at Yale or felt that I was being held back in some way. I chose not to leave because I felt very strongly that I wanted to be one of the women who persevered and pushed the door a little bit more open for the women coming behind me. The women who had come before me had had to do the harder work of getting the door to open just a crack.

1. Are there any professional directions that you regret not pursuing in your career?

I have absolutely no regrets. I love being a healthcare epidemiologist, it’s been a great career choice for me. I have watched some of my colleagues go on to be section chiefs, department heads, and chiefs of staff. Sometimes I thought maybe I should consider doing the same and pursue opportunities to climb the academic and administrative ladder, but realized I’m not really interested in that. At the end of my career at the VA, I was the acting chief of the infectious diseases section for 9 months, and that pretty much cured me of any inclination to do anything else besides healthcare epidemiology.

1. What are the top 3 emerging healthcare issues that leaders in epidemiology, stewardship, and our professional societies should keep their eye on over the next 5 years?

Artificial intelligence is a big one, which I know little about but am watching with interest from the sidelines. It’s becoming an important issue across healthcare including in healthcare epidemiology and stewardship. Ongoing issues with multidrug-resistant organisms and high consequence pathogens will continue to be global issues along with the challenges of maintaining preparedness for the next pandemic. Also, new technologies, new equipment and products related to patient care are being rapidly introduced without always understanding potential risks to patient safety. Another increasingly prevalent issue is the shortage and/or recall of products and equipment that requires us to adjust quickly to support patient care and balance that with patient safety. This necessitates the rapid dissemination of information and sharing with each other approaches to managing these challenging situations. This happened a lot during the COVID-19 pandemic and continues today.

1. Finally, to ensure the future of our workforce, how would you influence students and trainees to pursue infectious diseases, healthcare epidemiology or stewardship?

This ties in nicely to my SHEA lectureship talk, where I stated that we could do a better job advertising what we do and why we enjoy it so much. We’re still seen as an enigma. Nobody really understands what we do behind the scenes, including many of our ID colleagues. ID fellows may pursue a research project in healthcare epidemiology but not understand that there’s a whole career path here which can lead to major leadership roles. Likewise, engaging students about what we do during career panels is very important. I used to do this with high school students before the pandemic, sharing what I do and how I got involved with it and how my career has been a series of doing the things I said I would never do. Starting out, I was not going to be a doctor, and when I changed my mind, I had no intention of being a specialist, or a researcher, I was planning to be a general internist. I was never going to leave the Detroit area where I had grown up, gone to school, and did my medical training. I had a social network and my family there but the career opportunity to be a healthcare epidemiologist was not available in Detroit, so I moved my life to New Haven, Connecticut and Yale. I planned at the time to stay only two to three years, learn everything I could about healthcare epidemiology from my boss, Dr. Walter Hierholzer, then hopefully return to Michigan to a healthcare epidemiology position. That is not how things turned out and instead I built a fabulous career at Yale and a new life in Connecticut. So, my enduring advice to students and trainees is to be curious and open to possibilities and opportunities one might not have considered. We should encourage students and trainees to talk to us about our careers. We should be prepared for them to ask about what drew us to the field, why we like it, and what we don’t like or would do differently if we were to start over. And we should ask students and trainees “What are you thinking about? What are you interested in and why? Have you ever thought about ID? Have you considered healthcare epidemiology, or antimicrobial or diagnostic stewardship as a career path? Well, here are the opportunities in this career and this is why I am passionate about what I do. Here is how you can get started and I can help you if you are interested.” Maybe something from our personal career path will resonate with them, and they will find a passion they had not previously considered.

I am concerned that the COVID-19 pandemic might dissuade trainees from considering ID as a career path. It was not a short-lived crisis that we recovered from quickly. It lasted several years and took a toll on us. We were exhausted and did not always feel appreciated for our efforts and sacrifices. But as we emerge from this, I like to challenge colleagues to tell me at least one good thing personally and professionally that came from the pandemic. While putting the pandemic behind us, let’s not forget all the things we learned and did well, let’s keep showing our value and worth. Hopefully more trainees will catch our passion for ID, healthcare epidemiology and stewardship and they will want to join us and grow the next generation.

